# Supplementary material for: “You’re still worth it”: The moral and relational context of politically motivated unfriending decisions in online networks
Source: PLoS One. 2021 Jan 11;16(1):e0243049. doi: 10.1371/journal.pone.0243049 (PMC7799818; doi:10.1371/journal.pone.0243049)
Supplement: S1 File — (PDF) [file pone.0243049.s001.pdf]

Moral Unfriending: Study 1 and Study 2

Supplementary Analyses

November 2020

TABLE OF CONTENTS

A1. Overview of Hypotheses and Research Questions
A2. Study 1: Sample Description concerning Age
A3. Study 1: Sample Description concerning Gender
A4. Study 1: Sample Description concerning Political Ideology
A5. Study 1: Overview of Moral Foundations and Political Topics
A6. Study 1: Effects of Scenario Manipulation on Perceived Violations in Different Moral Domains
A7. Study 1: Psychometrics of Variables
A8. Study 2: Sample Description concerning Age
A9. Study 2: Sample Description concerning Gender
A10. Study 2: Sample Description concerning Political Ideology
A11. Study 2: Psychometrics of Variables
A12. Study 2: Results from the MANOVA Including Perceived Wrongness, Likelihood of Unfriending and Blocking as Dependent Variables
A13. Study 2: Structural Equation Model: Coefficients
References

A1. Overview of Hypotheses and Research Questions

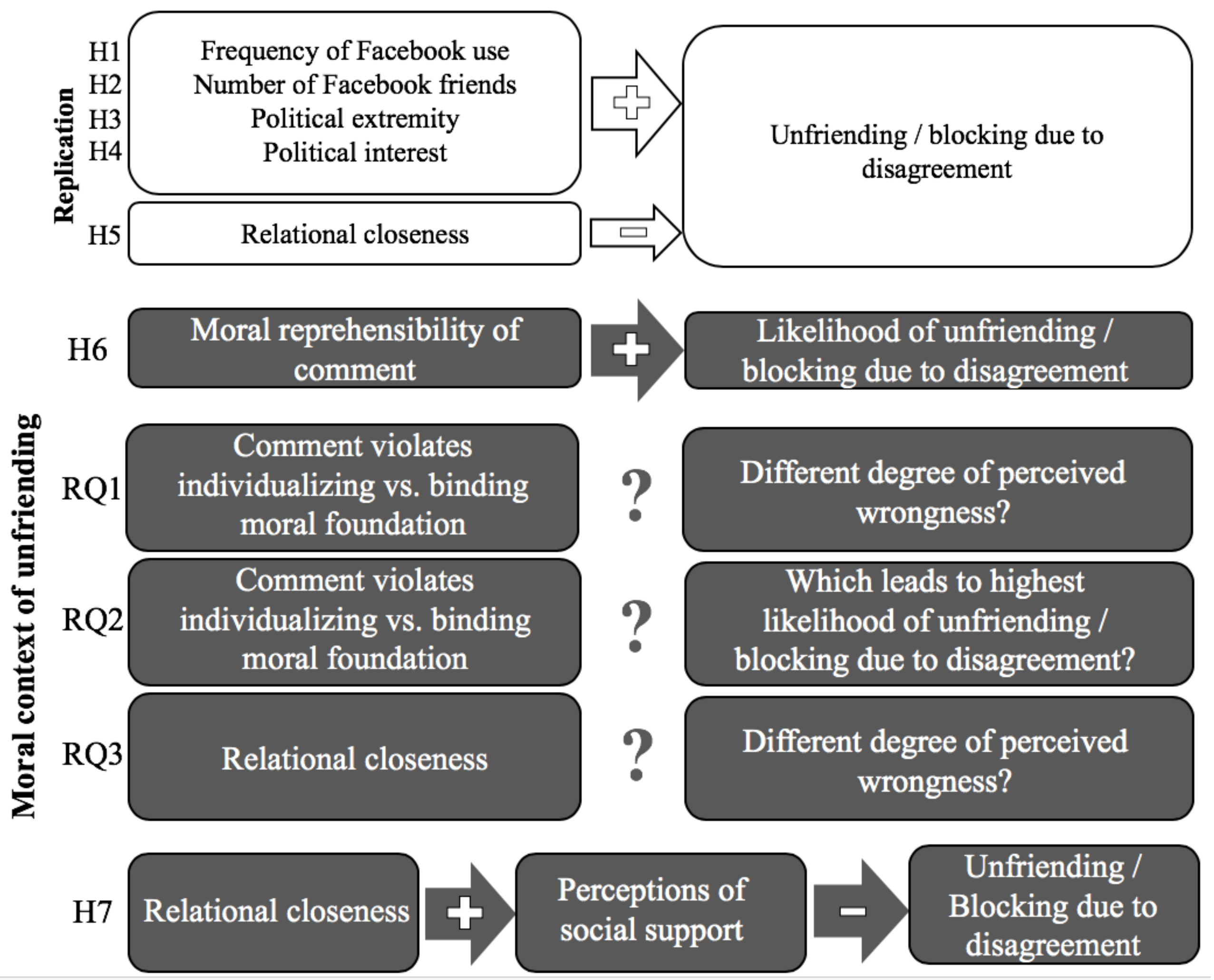

A2. Study 1: Sample Description concerning Age

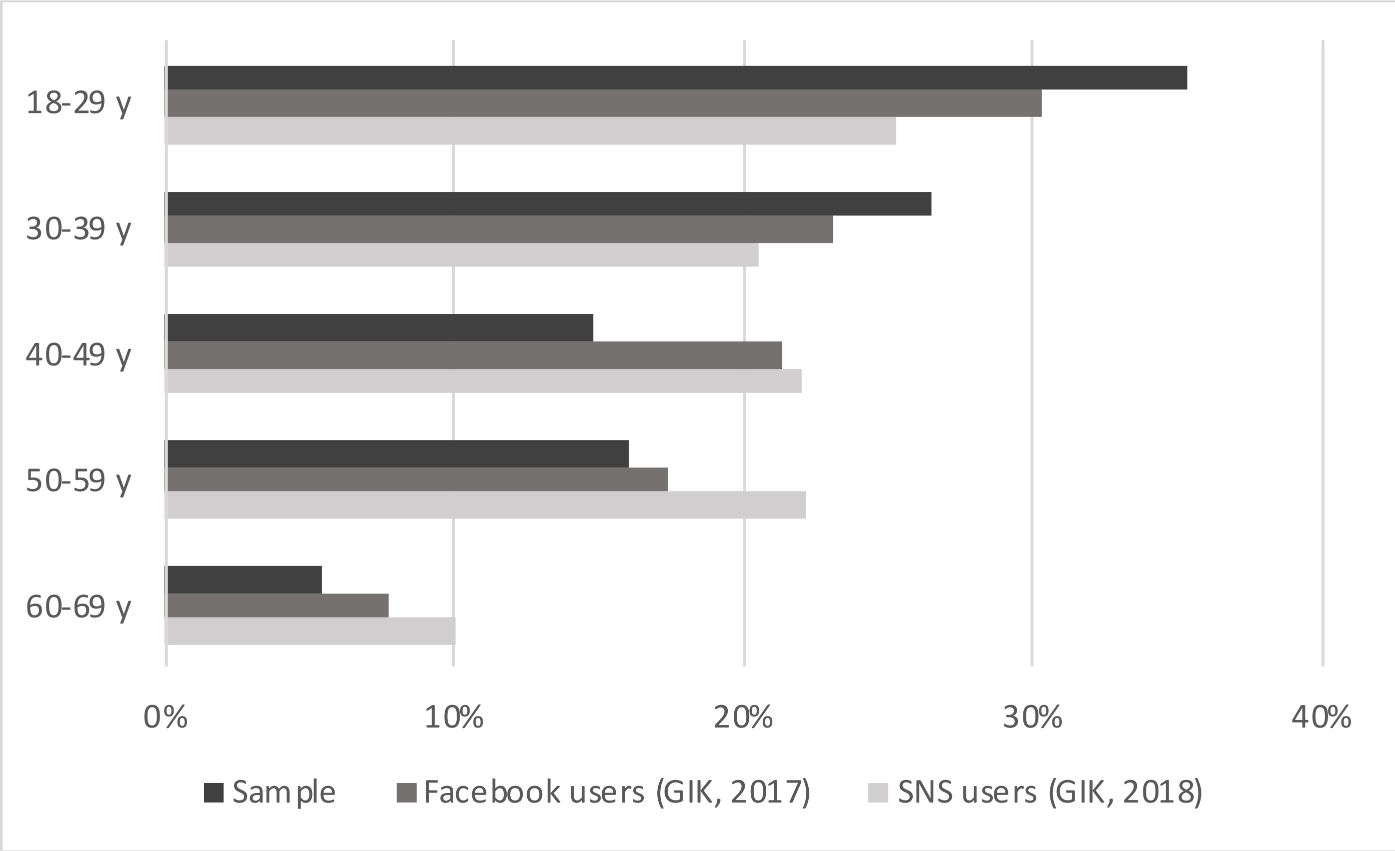

Please note that 1.8% of participants (N = 13) were older than 69 years. Those participants were excluded from this overview.

A3. Study 1: Sample Description concerning Gender

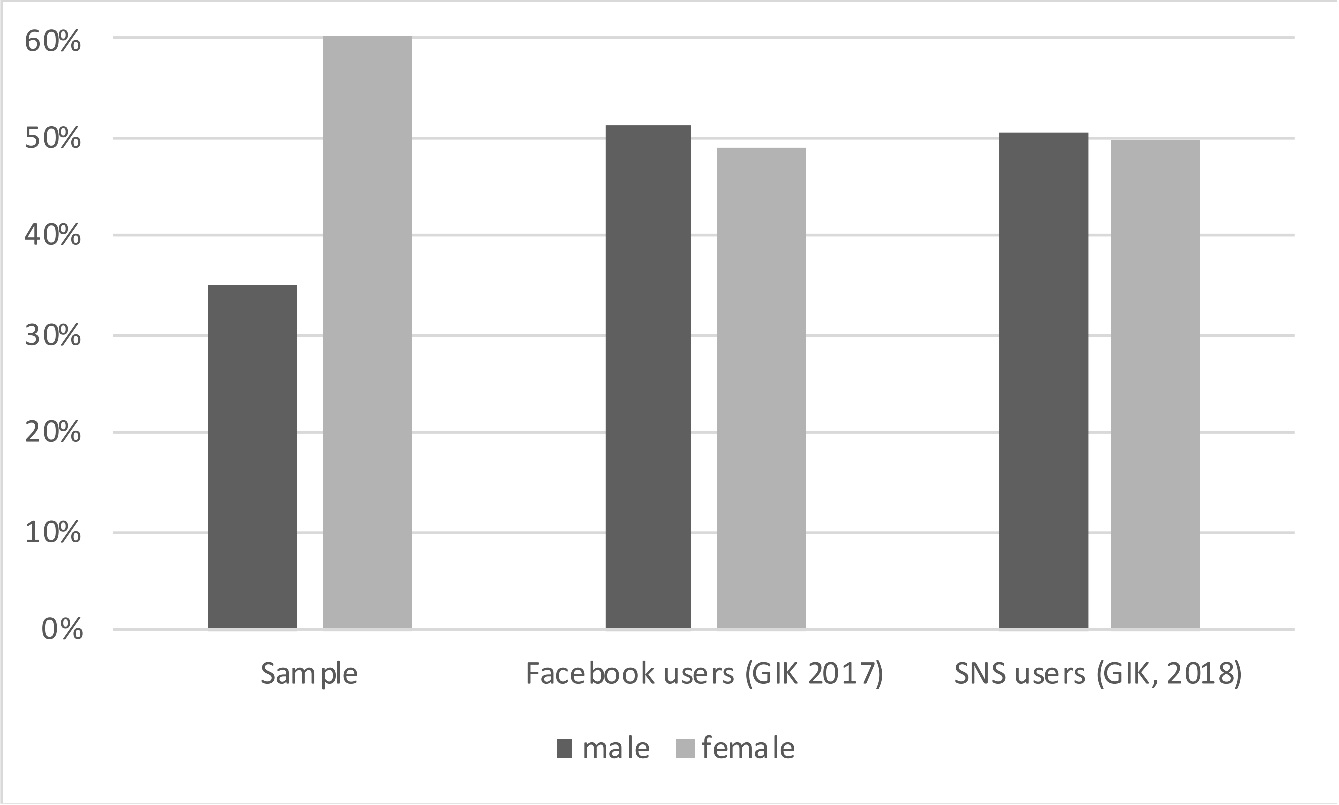

A4. Study 1: Sample Description concerning Political Ideology

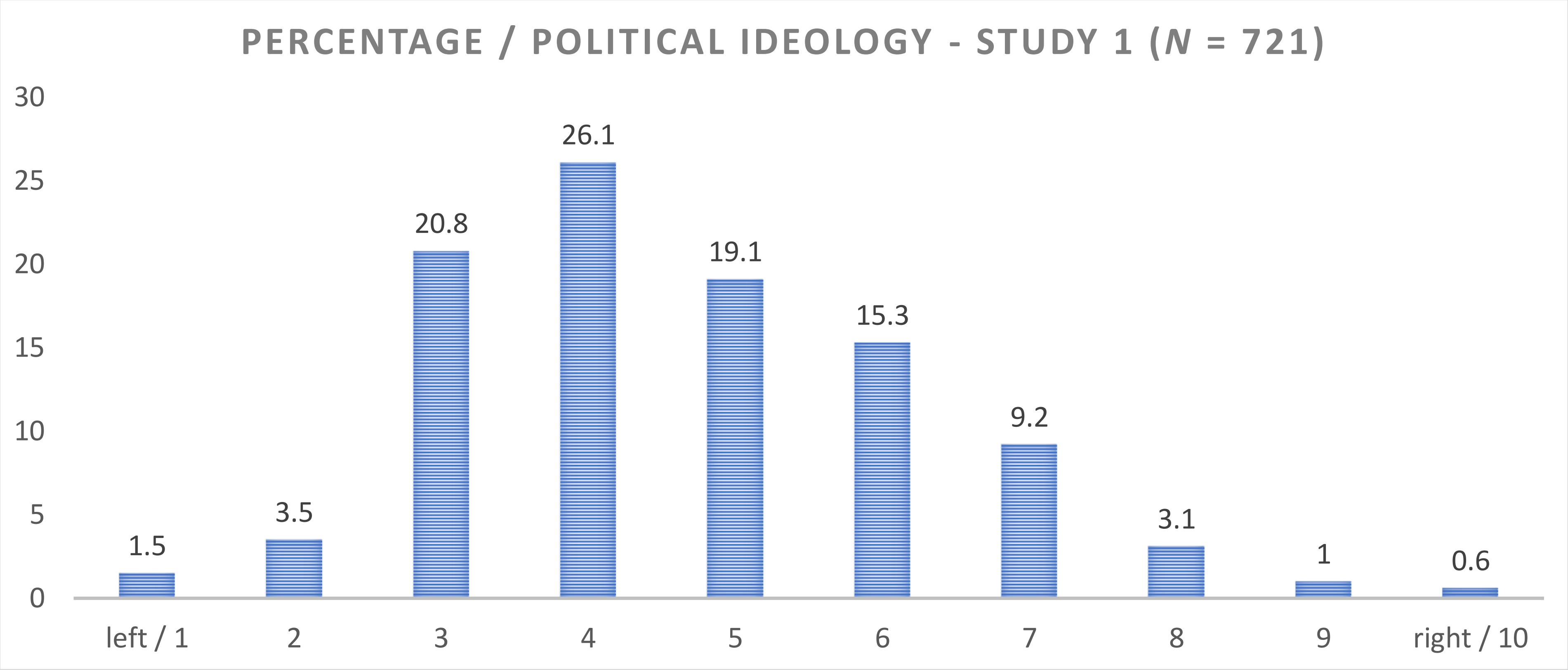

A5. Study 1: Overview of Moral Foundations and Political Topics (cf. MORAL UNFRIENDING 1 - STIMULUS AND QUESTIONNAIRE for further details)

Foundation	Political Topic
1 Harm/Care	War
2 Harm/Care	Refugees
3 Harm/Care	Famine
4 Fairness	Rich/Poor
5 Fairness	Gender Equality
6 Fairness	Religion Equality
7 Loyalty	German Patriotism
8 Loyalty	European Patriotism
9 Loyalty	Local Patriotism
10 Authority	Police
11 Authority	Government
12 Authority	Federal Intelligence Service
13 Purity	Pollution
14 Purity	Pornography
15 Purity	Prostitution

A6. Study 1: Effects of Scenario Manipulation on Perceived Violations in Different Moral Domains

	Moral Domain of Scenarios					Main effect		
Perceived violation in…	*Harm / Care*	*Fairness*	*Loyalty*	*Authority*	*Purity*	*F(4, 3600)*	*p*	η^2_p
Harm / Care	3.76 (1.30)$_a$	3.37 (1.31)$_b$	2.38 (1.28)$_c$	2.67 (1.34)$_d$	3.00 (1.48)$_e$	120.23	< .001	.12
Fairness	3.93 (1.25)$_a$	4.08 (1.16)$_a$	2.46 (1.30)$_b$	2.55 (1.34)$_{b,c}$	2.74 (1.43)$_c$	263.52	< .001	.23
Loyalty	2.44 (1.36)$_a$	2.80 (1.42)$_b$	3.19 (1.27)$_c$	2.47 (1.32)$_a$	2.38 (1.42)$_a$	45.14	< .001	.05
Authority	2.14 (1.23)$_{a,c}$	2.13 (1.18)$_{a,c}$	2.26 (1.22)$_c$	3.36 (1.38)$_b$	2.02 (1.24)$_a$	139.98	< .001	.14
Purity	2.69 (1.42)$_a$	2.25 (1.31)$_b$	1.87 (1.13)$_c$	1.94 (1.18)$_c$	2.69 (1.49)$_a$	64.49	< .001	.07

Note. Different subscripts in a row indicate significant differences with p < .05 using Bonferroni-corrected post hoc comparisons.

A7. Study 1: Psychometrics of Variables

	N	Min	Max	M	SD	Skewness	Kurtosis
Unfriending Likelihood (Mean of 5 scenarios)	721	1.00	5.00	2.7331	1.04218	.055	-.759
Frequency of FB Use	721	1	7	6.14	1.319	-1.748	2.409
Number of FB Friends	720	0	4500	243.16	316.743	6.629	69.477
Political Interest	721	1	5	3.85	.967	-.703	.060
Political Ideology (1 = left / 10 = right)	721	1	10	4.65	1.618	.464	.102
Political Extremity	721	1.00	5.00	2.0458	.97289	.743	.190
Perceived Wrongness of Message (Mean of 5 scenarios)	721	1.00	5.00	3.3842	.81665	-.649	.441
Personal Individualizing Foundation	721	1.42	6.00	4.8797	.59590	-1.141	2.998
Personal Binding Foundation	721	1.00	5.33	3.3564	.71614	-.081	-.187

A8. Study 2: Sample Description concerning Age

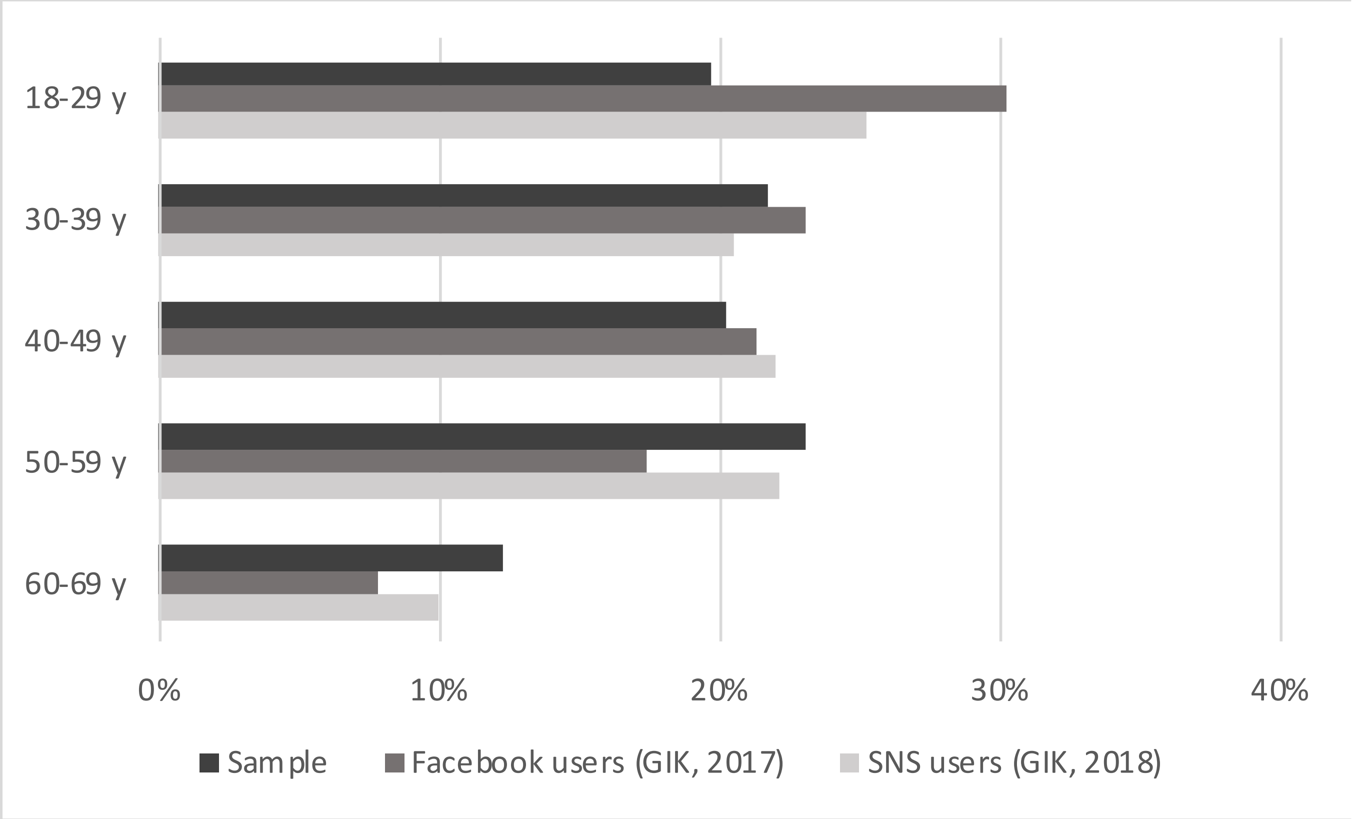

Please note that 2.92% of participants (N = 24) were older than 69 years. Those participants were excluded from this overview.

A9. Study 2: Sample Description concerning Gender

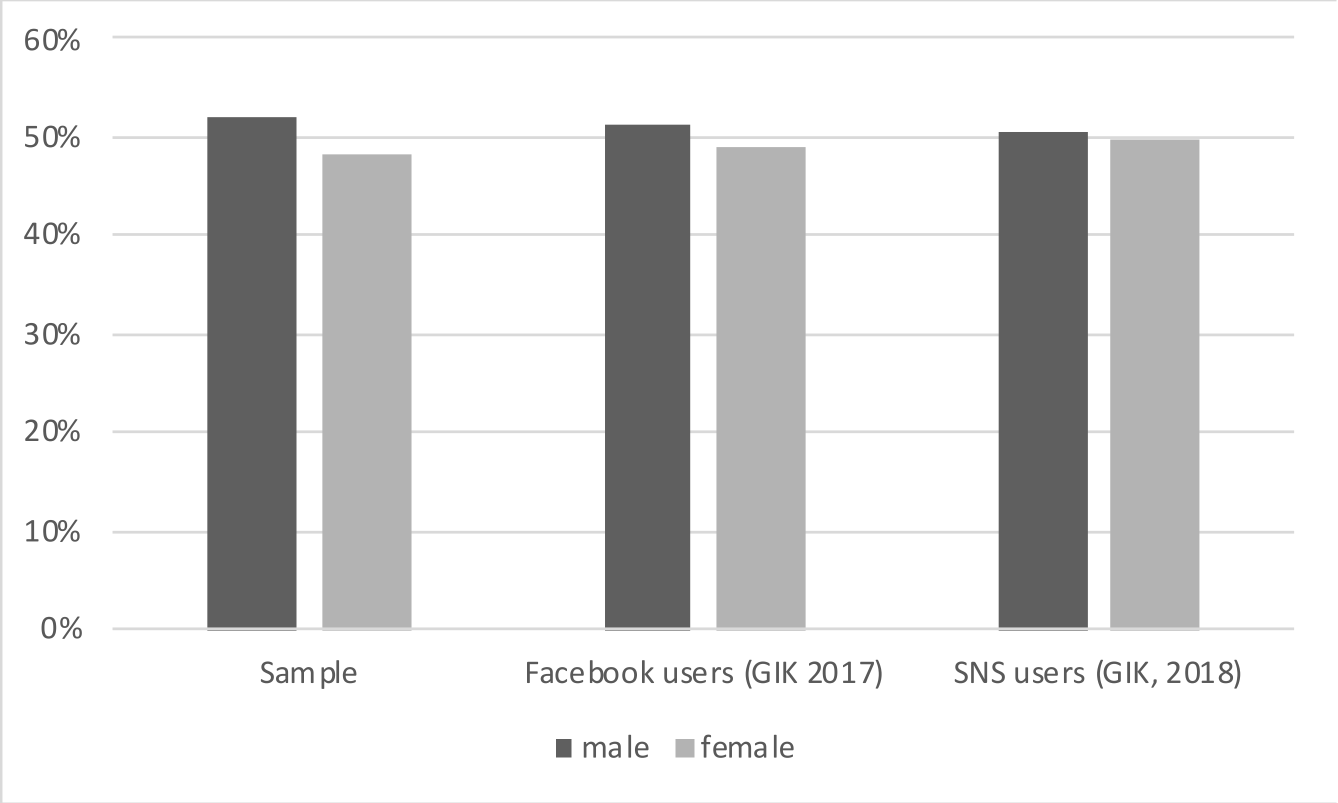

A10. Study 2: Sample Description concerning Political Ideology

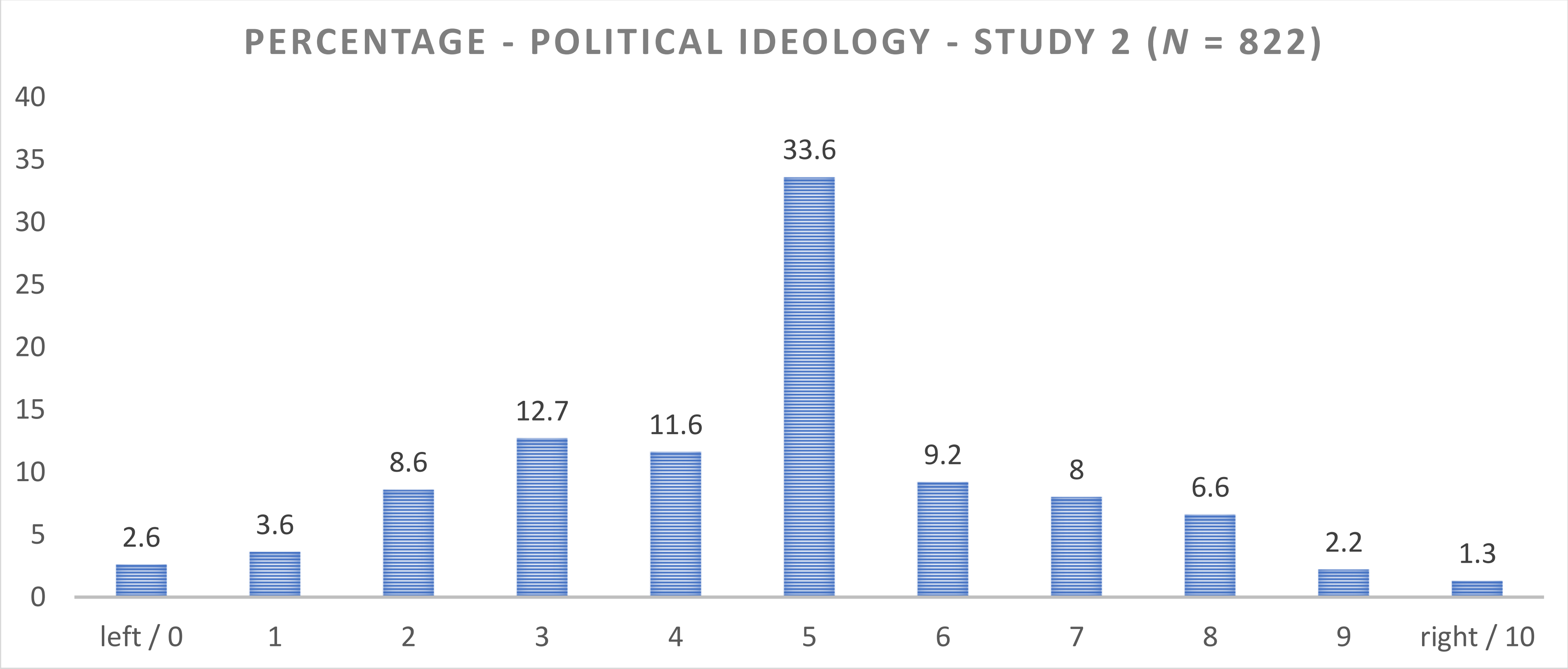

A11. Study 2: Psychometrics of Variables

	N	Min	Max	M	SD	Skewness	Kurtosis
Unfriending Likelihood	822	1	5	2.64	1.250	.287	-.926
Blocking Likelihood	822	1	5	2.64	1.291	.318	-.955
Frequency of FB Use	822	2	7	6.27	1.072	-1.781	3.155
Number of FB Friends	822	1	3000	187.88	289.432	5.098	35.889
Political Interest	822	1	5	3.66	.976	-.577	.308
Political Ideology (0 = left / 10 = right)	822	1	11	5.70	2.056	.044	-.003
Political Extremity	822	1.00	6.00	2.5061	1.43026	.660	-.463
Perceived Wrongness of Status Update	822	1	5	3.20	1.171	-.204	-.719
Perceived Violation of Individualizing Foundation	822	1.00	5.00	2.9337	1.12908	.028	-.842
Perceived Violation of Binding Foundation	822	1.00	5.00	2.6979	1.00384	.184	-.527
Informational Support	822	1.00	5.00	3.1054	1.00894	-.173	-.497
Emotional Support	822	1.00	5.00	3.2137	1.14133	-.203	-.850
Instrumental Support	822	1.00	5.00	3.1237	1.18454	-.156	-.881
Relationship Satisfaction	822	1.00	6.00	4.9585	.65919	-1.442	5.182
Personal Individualizing Foundation	822	1.00	6.00	3.9961	.72826	-.319	.963

A12. Study 2: Results from the MANOVA Including Perceived Wrongness, Likelihood of Unfriending and Blocking as Dependent Variables

Effect	*Wilks'λ*	*F*	*df*	*p*	η_p^2
**Perceived opinion climates**					
Relational Closeness	0.98	2.78	6,1628	.011	.01
Moral Foundation	0.99	2.51	3,814	.058	.01
Relational Closeness x Moral Foundation	0.99	2.00	6,1628	.063	.01

A13. Study 2: Structural Equation Model: Coefficients

	β	b [95% CI]	Std.Err	z-value	P(>|z|)
Informational Support ~ Relational Closeness (a1)	0.565	1.277 [1.109, 1.445]	0.086	14.874	0.000
Emotional Support ~ Relational Closeness (a2)	0.598	1.497 [1.316, 1.678]	0.092	16.195	0.000
Instrumental Support ~ Relational Closeness (a3)	0.576	1.396 [1.213, 1.578]	0.093	14.964	0.000
Unfriending ~ Informational Support (b1)	0.249	0.282 [-0.027, 0.591]	0.158	1.786	0.074
Unfriending ~ Emotional Support (b2)	-0.413	-0.421 [-0.786, -0.056]	0.186	-2.260	0.024
Unfriending ~ Instrumental Support (b3)	0.033	0.035 [-0.255, 0.324]	0.148	0.235	0.814
Unfriending ~ Relational Closeness (d1)	-0.070	-0.179 [-0.443, 0.085]	0.135	-1.331	0.183
Blocking ~ Informational Support (c1)	0.159	0.184 [-0.141, 0.509]	0.166	1.110	0.267
Blocking ~ Emotional Support (c2)	-0.265	-0.276 [-0.652, 0.101]	0.192	-1.435	0.151
Blocking ~ Instrumental Support (c3)	-0.011	-0.011 [-0.302, 0.279]	0.148	-0.077	0.938
Blocking ~Relational Closeness (d2)	-0.064	-0.167 [-0.433, 0.098]	0.135	-1.237	0.216

Note. Explained variation: Informational Support: $R^2 = 0.320$; Emotional Support: $R^2 = 0.357$; Instrumental Support: $R^2 = 0.332$; Unfriending: $R^2 = 0.052$; Blocking: $R^2 = 0.034$.

References

Gesellschaft für Integrierte Kommunikationsforschung (GIK) (2017). Best for Planning 2017. https://gik.media/best-4-planning

Gesellschaft für Integrierte Kommunikationsforschung (GIK) (2018). Best for Planning 2018. https://gik.media/best-4-planning
